# IVUS-optimized sequential rotational atherectomy with IABP support for severely calcified unprotected left main disease: a systematic integration strategy: a case report

**DOI:** 10.1093/ehjcr/ytag294

**Published:** 2026-04-25

**Authors:** Jianping Liu, Fuhai Li, Zijing Jiang, Ling Liu, Chun Xiao

**Affiliations:** Department of Cardiology, the Third People's Hospital of Huizhou and the Affiliated Hospital of Guangzhou Medical University, Huizhou City, Guangdong Province, 516002, China; Department of Cardiology, the Third People's Hospital of Huizhou and the Affiliated Hospital of Guangzhou Medical University, Huizhou City, Guangdong Province, 516002, China; Department of Cardiology, the Third People's Hospital of Huizhou and the Affiliated Hospital of Guangzhou Medical University, Huizhou City, Guangdong Province, 516002, China; Department of Cardiology, the Third People's Hospital of Huizhou and the Affiliated Hospital of Guangzhou Medical University, Huizhou City, Guangdong Province, 516002, China; Department of Cardiology, the Third People's Hospital of Huizhou and the Affiliated Hospital of Guangzhou Medical University, Huizhou City, Guangdong Province, 516002, China

**Keywords:** unprotected left main, Rotational atherectomy, Intravascular ultrasound, Mechanical circulatory support, Coronary calcification, Hibernating myocardium, Case report

## Abstract

**Background:**

Severely calcified unprotected left main coronary artery disease with Grade IV calcification (360-degree circumferential arc) and prohibitive surgical risk represents an extreme-risk scenario lacking standardized management approaches.

**Case summary:**

A 72-year-old diabetic male (EuroSCORE II 8.2%) with progressive angina underwent systematic six-stage percutaneous coronary intervention integrating: (1) bilateral iliac stenting for access preparation; (2) prophylactic intra-aortic balloon pump; (3) pre-procedural IVUS-characterized sequential rotational atherectomy (1.25→1.5 mm burrs) with angiography-guided burr upgrade and post-atherectomy IVUS confirmation; (4) systematic lesion preparation; (5) drug-eluting stent implantation; and (6) aggressive post-dilation achieving IVUS-optimized minimal stent area 9.39 mm^2^ (244% increase from baseline 2.73 mm^2^; maximal stent area 14.16 mm^2^, 419% increase). Ten-month follow-up demonstrated remarkable functional recovery with left ventricular ejection fraction improvement from 47% to 66% (40% relative increase), confirming hibernating myocardium revascularization with sustained symptom freedom.

**Discussion:**

This case demonstrates that systematic integration of underrecognized access vessel preparation, mechanical circulatory support, strategic IVUS utilization at critical decision points, and evidence-driven optimization achieves excellent acute and long-term outcomes in Grade IV calcified unprotected left main disease. The reproducible six-stage framework expands treatment options for previously inoperable high-risk patients, with objective functional recovery validating aggressive intervention when surgical revascularization is contraindicated.

Learning points

- A systematic six-stage integration strategy (access to optimization) achieves excellent outcomes in inoperable Grade IV calcified left main disease.
- Strategic IVUS utilization at critical decision points enables rational sequential burr selection and aggressive post-dilation without requiring imaging at every step.
- Objective functional recovery at 10 months confirms that revascularizing hibernating myocardium provides durable clinical benefit in high-risk patients.

## Introduction

Unprotected left main coronary artery disease with Grade IV calcification (360-degree circumferential arc) represents an extreme-risk scenario combining anatomic complexity and procedural difficulty.^1,2^ While coronary artery bypass grafting remains guideline-recommended first-line therapy,^3^ approximately 20%–30% of patients present with prohibitive surgical risk.^4^ For these patients, percutaneous coronary intervention (PCI) offers the only revascularization option, yet severe circumferential calcification prevents adequate stent expansion—the primary predictor of stent thrombosis and restenosis.^5–7^

Successful outcomes require integration of rotational atherectomy for calcium modification,^8,9^ intravascular ultrasound (IVUS) for lesion characterization and optimization,^10,11^ and mechanical circulatory support for hemodynamic protection.^12^ We present a case demonstrating a comprehensive six-stage strategy, emphasizing that the novelty lies in the structured sequencing and integration of these established techniques to achieve optimal mechanical results and remarkable long-term functional recovery at 10-month follow-up.

## Summary figure

**Figure:**
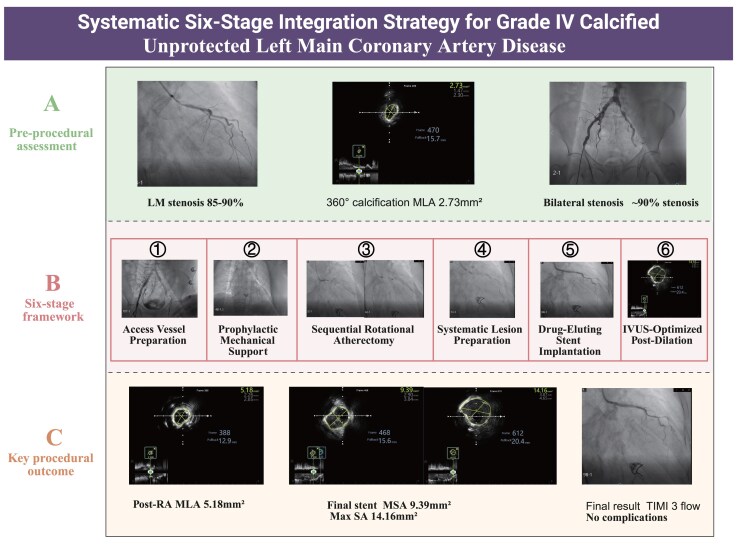


## Case Summary

### Patient information and clinical findings

A 72-year-old male presented with Canadian Cardiovascular Society class III angina worsening over one month. His cardiovascular risk profile included 15-year diabetes mellitus (HbA1c 8.2%), hypertension, 30-year smoking history, and extensive prior revascularization. Bilateral upper extremity access was compromised by previous interventions, mandating a transfemoral approach.

Transthoracic echocardiography revealed a left ventricular ejection fraction of 47% with regional hypokinesis.EuroSCORE II was 8.2% and STS predicted mortality of 5.2%. The heart team consensus determined that PCI with comprehensive mechanical support as optimal strategy.

### Physical examination

On admission, the patient was haemodynamically stable. Blood pressure was 138/82 mmHg (normal: 90–120/60–80 mmHg), heart rate was 76 beats per minute (normal: 60–100 bpm), respiratory rate was 16 breaths per minute (normal: 12–20 breaths/min), and oxygen saturation was 99% on room air (normal: ≥95%). Cardiovascular examination revealed a regular heart rhythm with normal S1 and S2 heart sounds, no murmurs, rubs, or gallops. Jugular venous pressure was not elevated. Peripheral pulses were symmetrically diminished bilaterally in the upper extremities, consistent with prior vascular interventions. Mild bilateral ankle oedema (1+) was noted. Chest auscultation revealed clear lung fields bilaterally without crepitations or wheeze. There were no signs of acute heart failure or haemodynamic compromise on presentation.

### Diagnostic assessment

**Figure 1:**
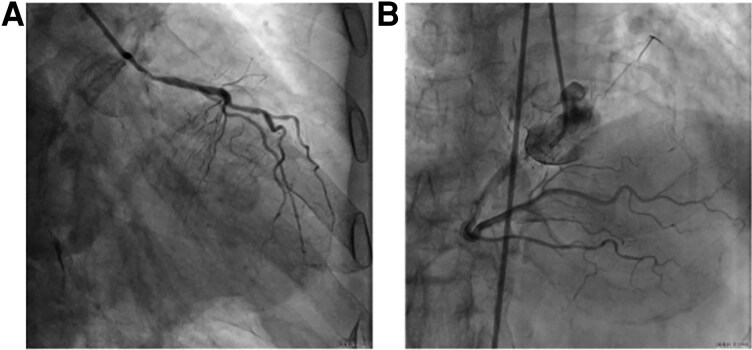
Baseline diagnostic coronary angiography. (*A*) Right anterior oblique caudal projection demonstrating severe diffuse stenosis of the left main coronary artery extending from ostium through entire trunk to distal bifurcation, with angiographically visible calcification throughout the lesion. (*B*)The right coronary artery provides robust collateral circulation (Rentrop grade 2–3) to the left circumflex territory.

**Figure 2:**
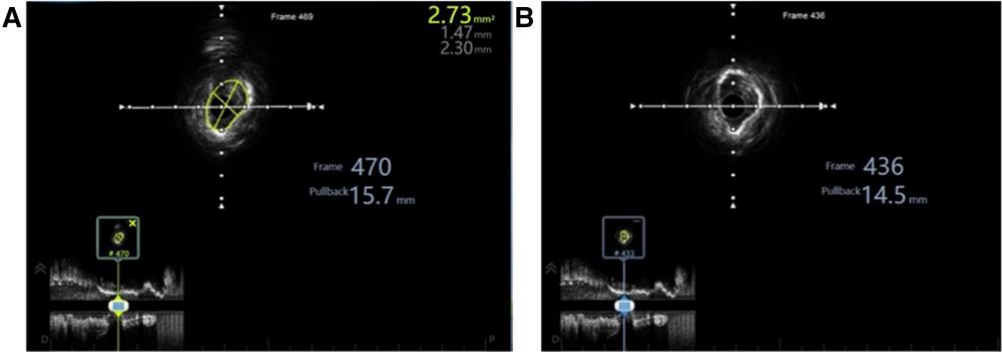
Intravascular ultrasound assessment of left main coronary artery calcification. (*A*) Pre-procedural IVUS cross-section at the most stenotic segment of the left main trunk demonstrating a minimal luminal area of 2.73 mm^2^ (traced in yellow), representing severe flow limitation. Normal reference left main area typically ranges from 15–20 mm^2^. (*B*) Pre-procedural IVUS cross-section showing 360-degree calcification arc, classifying this as Grade IV calcification according to established criteria.

Coronary angiography revealed severe diffuse 85%–90% left main stenosis extending from ostium to distal bifurcation (*Figure 1A* and *B*, Supplementary material online, *Video S1*–S2). Left anterior descending ostium demonstrated 70–80% stenosis with patent previous stents distally. Left circumflex artery showed chronic total occlusion beyond ostium with robust collaterals from the dominant right coronary artery (Rentrop grade 2–3). Bilateral iliac angiography demonstrated severe 90% stenosis necessitating initial treatment before coronary intervention (see Supplementary material online, *Figure S1*, Supplementary material online, *Video S3*). IVUS examination revealed a left main minimal luminal area of 2.73 mm^2^ with 360-degree circumferential calcification (Grade IV) predominantly superficial (*Figure 2*,Supplementary material online, *Video S4*).

Pre-procedural laboratory investigations demonstrated: haemoglobin 128 g/l (normal: 130–175 g/l), haematocrit 38.5% (normal: 40–52%), platelet count 187 × 10^9^/l (normal: 150–400 × 10^9^/l), white blood cell count 7.2 × 10^9^/l (normal: 4.0–10.0 × 10^9^/l). Metabolic panel revealed serum creatinine 124 μmol/l (normal: 62–106 μmol/l) with estimated glomerular filtration rate 52 ml/min/1.73 m^2^ (normal: ≥60 ml/min/1.73 m^2^), consistent with CKD stage 3a. Fasting blood glucose was 9.8 mmol/l (normal: 3.9–6.1 mmol/l) and HbA1c 8.2% (normal: <7.0% for diabetic management target). Lipid profile: total cholesterol 4.8 mmol/l (normal: <5.2 mmol/l), LDL-C 2.9 mmol/l (normal: <1.8 mmol/l for high-risk patients), HDL-C 0.9 mmol/l (normal: >1.0 mmol/l), triglycerides 2.1 mmol/l (normal: <1.7 mmol/l). High-sensitivity C-reactive protein was 4.2 mg/l (normal: <3.0 mg/l). NT-proBNP was 683.2 pg/ml (normal: <125 pg/ml). High-sensitivity troponin I was within normal limits pre-procedurally (<0.04 ng/ml; normal: <0.04 ng/ml). Coagulation: prothrombin time 11.8 s (normal: 11–13 s), international normalized ratio 1.0 (normal: 0.9–1.1).

### Six-stage systematic integration strategy

**Stage 1: Access Vessel Preparation.** Sequential balloon angioplasty followed by 8.0 mm × 100 mm self-expanding stent deployment bilaterally achieved excellent iliac patency (see Supplementary material online, *Figure S2*).

**Stage 2: Prophylactic Mechanical Support.** A 40 cc intra-aortic balloon pump was inserted via the left femoral artery, achieving diastolic augmentation of 22 mmHg and systolic unloading of 15 mmHg.IABP was selected over larger-bore mechanical support (e.g. Impella) due to the severe iliac artery stenosis and freshly deployed stents, which contraindicate large-bore sheath insertion.

**Figure 3:**
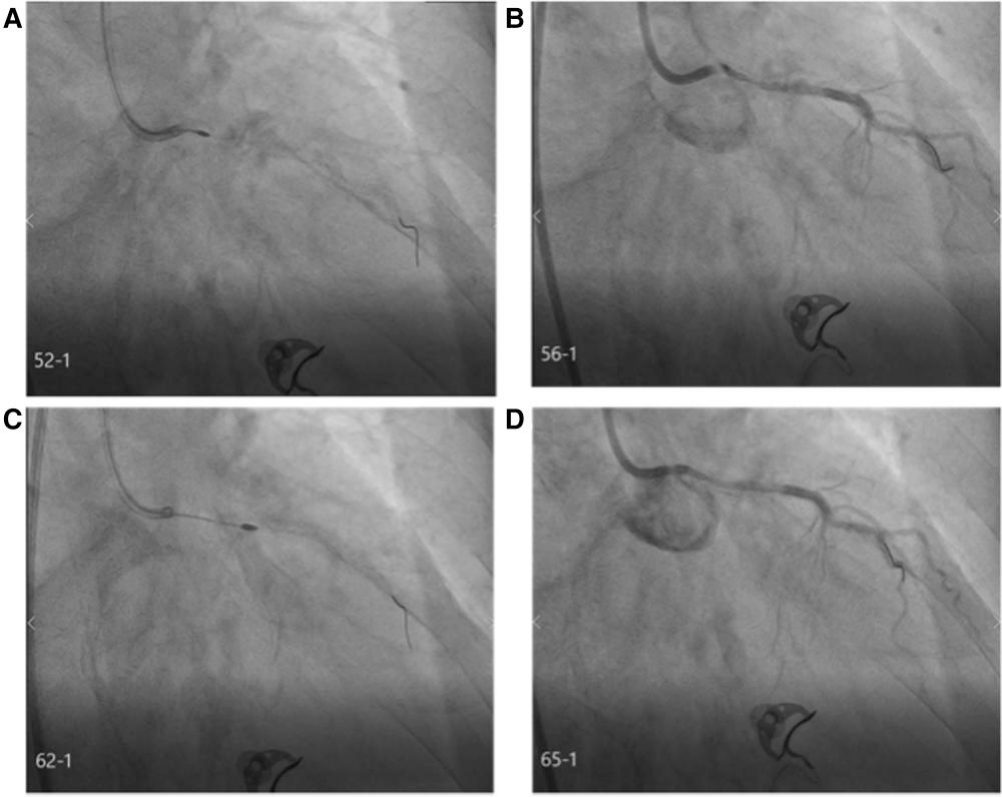
Sequential rotational atherectomy procedure. (*A*) Fluoroscopic image of the 1.25 mm rotational atherectomy burr positioned in the left main coronary artery. (*B*) Coronary angiography immediately after 1.25 mm burr atherectomy, demonstrating TIMI grade 3 flow but persistent severe stenosis with limited luminal gain. (*C*) Fluoroscopic image of the 1.5 mm rotational atherectomy burr positioned in the left main coronary artery after burr upgrade decision. (*D*) Coronary angiography immediately after 1.5 mm burr atherectomy demonstrated significantly improved luminal dimensions with substantial reduction in angiographic stenosis severity and maintained TIMI grade 3 flow.

**Figure 4:**
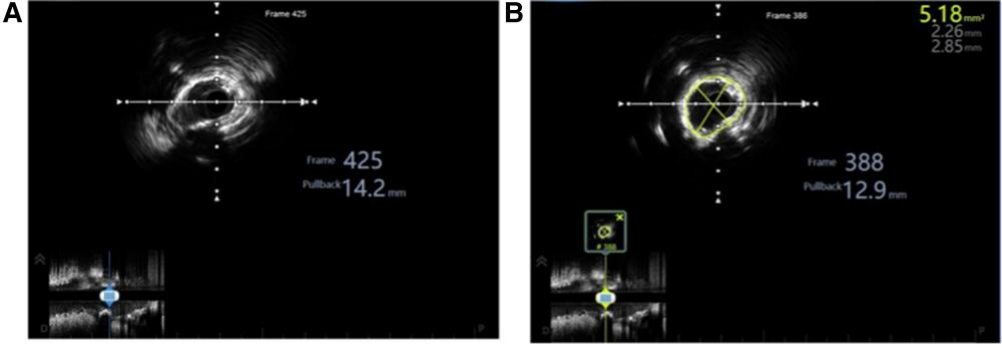
Post-rotational atherectomy IVUS assessment confirming calcium modification. (*A*) IVUS cross-section after 1.5 mm burr rotational atherectomy demonstrating extensive calcium fracture with multiple reverberating reflection artifacts. (*B*) IVUS cross-section after rotational atherectomy showing improved minimal luminal area of 5.18 mm^2^ (traced in yellow), representing 90% increase from baseline 2.73 mm^2^.

**Stage 3: Sequential Rotational Atherectomy.** Initial 1.25 mm burr atherectomy at 160 000 rpm was performed with meticulous technique: gentle pecking advancement, continuous RotaGlide infusion, run duration <30 s (*Figure 3A*, Supplementary material online, *Video S6*).Post-1.25 mm angiography showed persistent severe stenosis (*Figure 3B*, Supplementary material online, *Video S7*), prompting upgrade to 1.5 mm burr (*Figure 3C*, Supplementary material online, *Video S8*). Post-1.5 mm burr angiography demonstrated significantly improved luminal dimensions (*Figure 3D*, Supplementary material online, *Video S9*). IVUS confirmed achievement of atherectomy endpoints: extensive calcium fracture with multiple reflection artefacts, improved minimal luminal area to 5.18 mm^2^ (90% increase from baseline), no dissection or perforation (*Figure 4A* and *B*, Supplementary material online, *Video S10*).

**Stage 4: Systematic Lesion Preparation.** Progressive balloon predilation using 2.5 mm × 15 mm non-compliant balloon, followed by 3.0 mm × 6 mm cutting balloon, created controlled calcium incisions.

**Stage 5: Drug-Eluting Stent Implantation.** A 3.5 mm × 23 mm everolimus-eluting stent was deployed covering the left main body to the proximal left anterior descending artery (see Supplementary material online, *Figure S3*, Supplementary material online, *Video S11*).

**Figure 5:**
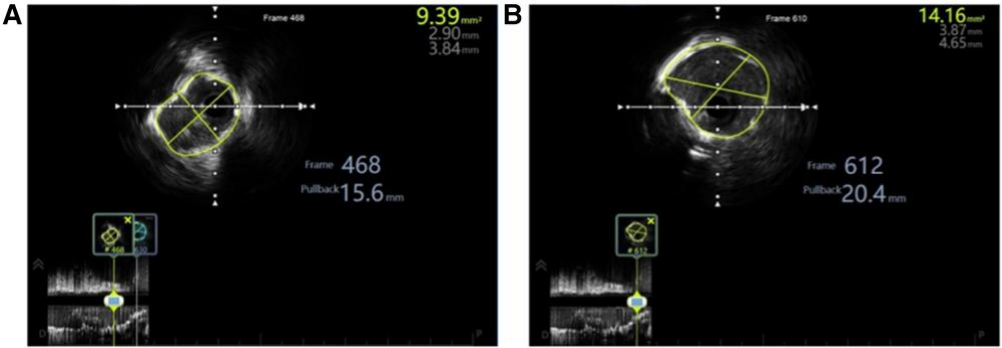
Final IVUS assessment demonstrating optimal stent expansion after systematic staged high-pressure post-dilation. (*A*) Final IVUS cross-section at the segment with minimal stent area demonstrating 9.39 mm^2^ (traced in green outline), substantially exceeding the Kang criteria (≥8 mm^2^ for left main body). (*B*) Final IVUS cross-section at the site of maximal stent area showing 14.16 mm^2^ (traced in green) with symmetric stent expansion and optimal geometric configuration, representing 419% increase from baseline 2.73 mm^2^.

**Stage 6: IVUS-Optimized Post-Dilation.** Staged high-pressure post-dilation (up to 18 atmospheres) was performed. Final IVUS confirmed minimal stent area 9.39 mm^2^, maximal stent area 14.16 mm^2^ (244% and 419% increase from baseline 2.73 mm^2^, respectively), with complete apposition, and no edge dissection, expansion ratio >0.80 (*Figure 5A* and *B*, Supplementary material online, *Video S12*).^13^

Final angiography demonstrated excellent patency with TIMI 3 flow and patent left circumflex ostium (see Supplementary material online, *Figure S4*, Supplementary material online, *Video S13*). IABP was systematically weaned and removed without complication. Total procedure: 180 min, fluoroscopy 45 min, contrast 280 ml, radiation 2850 mGy. Given the contrast load and CKD stage 3a, strict hydration protocols (1 ml/kg/h saline) were maintained. Radiation exposure was minimized through low-frame rate fluoroscopy and collimation. No complications occurred.

### Outcome and follow-up

Peak troponin I was 2.8 ng/ml (mild periprocedural myocardial injury). Patient was discharged on day 3 with dual antiplatelet therapy (aspirin plus ticagrelor), high-intensity lipid-lowering therapy (atorvastatin plus evolocumab), sacubitril/valsartan, and metoprolol succinate. During the hospital stay, medications included: unfractionated heparin intravenous infusion titrated to achieve activated clotting time 250–300 s during the procedure; aspirin 300 mg loading dose followed by 100 mg once daily (ongoing); ticagrelor 180 mg loading dose followed by 90 mg twice daily (ongoing, minimum 12 months); intravenous RotaGlide flush solution (glyceryl trinitrate 50 mcg/ml plus verapamil 0.1 mg/ml) continuously throughout rotational atherectomy for vasospasm prevention; intravenous adenosine 90 mcg boluses as needed for slow-flow prevention; intravenous normal saline 1 ml/kg/h for contrast-induced nephropathy prophylaxis initiated 12 h pre-procedure and continued 12 h post-procedure. On discharge: aspirin 100 mg once daily (lifelong); ticagrelor 90 mg twice daily (minimum 12 months); atorvastatin 40 mg once daily; evolocumab 140 mg subcutaneously every 2 weeks; sacubitril/valsartan 50 mg twice daily; metoprolol succinate 47.5 mg once daily.

At 10-month follow-up, the patient demonstrated complete angina freedom (NYHA class I). Left ventricular ejection fraction improved to 66% (from 47%), representing 40% relative improvement, with complete normalization of wall motion (wall motion score index 1.4→1.0) and significant reverse remodelling (left ventricular end-diastolic diameter 48→36 mm), confirming hibernating myocardium revascularization. NT-proBNP decreased 44% (683.2→385 pg/ml) and LDL-C achieved the guideline target (1.2 mmol/l).

## Discussion

This case demonstrates that systematic integration of advanced techniques achieves excellent acute and sustained outcomes in Grade IV calcified unprotected left main disease.

While individual techniques are established, the optimal integration sequence remains poorly defined. Our six-stage framework provides a reproducible approach addressing all procedural complexity components. Initial bilateral iliac stenting, though adding 25 min, was essential, as inadequate access preparation could result in IABP insertion failure or inability to advance rotational atherectomy system. Access vessel assessment represents an underrecognized critical component of high-risk PCI planning.

Strategic IVUS utilization at critical decision points—rather than routine serial imaging—maintained excellent outcomes while reducing procedure time. Pre-procedural IVUS characterized Grade IV calcification，informing a sequential burr strategy. Post-atherectomy IVUS confirmed adequate calcium fracture and final IVUS verified optimal outcomes: MSA 9.39 mm^2^, substantially exceeding Kang criteria (≥8 mm^2^)^14^ and approaching contemporary optimal targets.^15^

The aggressive post-dilation strategy reflects an understanding that adequate stent expansion is the most important long-term outcome predictor in left main PCI.^7,13,14^ Importantly, aggressive post-dilation was safe specifically because rotational atherectomy created extensive calcium fracture—attempting similar pressures in unmodified calcified lesions would risk perforation.

The remarkable 40% relative LVEF improvement (47%→66%) at 10-month follow-up, accompanied by complete, significant reverse remodelling, provides compelling evidence of hibernating myocardium recovery,^15^ validating clinical benefit beyond symptom relief. Our approach aligns with ESC/EACTS Guidelines, recognizing PCI as a class IIa recommendation for unprotected left main disease when surgery carries prohibitive risk.^14^

## Clinical implications

This systematic integration framework is generalizable to other extreme-risk scenarios, including bifurcation lesions with severe calcification, chronic total occlusions with heavy calcium burden, and calcified ostial lesions. The reproducible approach expands treatment options for patients previously considered inoperable or suitable only for palliative care. Sustained objective functional recovery with hibernating myocardium revascularization at 10-month follow-up demonstrates that with appropriate patient selection, meticulous procedural planning, and optimal technical execution, excellent durable outcomes can be achieved in the most challenging coronary interventions. However, while the conceptual planning framework is broadly applicable, the technical execution of high-speed rotational atherectomy and interpretation of IVUS-guided optimization requires advanced operator expertise typical of high-volume tertiary centres.

## Conclusions

Sequential rotational atherectomy with strategic IVUS utilization, integrated within a comprehensive six-stage systematic framework, achieves excellent acute and sustained 10-month outcomes in Grade IV calcified unprotected left main disease. Remarkable sustained functional recovery (LVEF 47%→66%) validates aggressive intervention in carefully selected high-risk patients, expanding therapeutic options for this vulnerable population.

## Patient perspective

After years of progressive chest pain limiting my daily activities, I was told surgery was too risky. The interventional team offered me this complex procedure with a detailed explanation of risks and benefits. Ten months later, I remain completely free of chest pain and have returned to an active life. This treatment gave me hope when I thought I had no options left.

## Supplementary Material

ytag294_Supplementary_Data

## Data Availability

All data underlying this article are available in the article and in its online Supplementary material. Additional anonymized data are available from the corresponding authors upon reasonable request.

## Lead author biography

Dr. Liu Jianping is an Associate Chief Physician and head of the Cardiovascular Medicine Department at Huizhou Third People's Hospital. With over 20 years of experience in cardiology, he specializes in coronary and peripheral vascular interventions. He graduated from the Clinical Medicine program at Wuhan University in July 2003 and has worked at Yichang Gezhouba Central Hospital before joining Huizhou Third People's Hospital in December 2012. He is an active member of the Guangdong Provincial Society of Integrative Medicine and the Interventional Cardiovascular Society, focusing on advancing interventional techniques and patient safety.

## Notes

**Consent:** Written informed consent was obtained from the patient for publication of this case report, accompanying images, and procedural videos, including sharing on social media platforms. The patient was shown the manuscript prior to submission. A copy of the written consent is available for review by the Editor-in-Chief of this journal on request.
