# Supplementary figures and images for: IVUS-optimized sequential rotational atherectomy with IABP support for severely calcified unprotected left main disease: a systematic integration strategy: a case report
Source: Eur Heart J Case Rep. 2026 Apr 25;10(5):ytag294. doi: 10.1093/ehjcr/ytag294 (PMC13188154; doi:10.1093/ehjcr/ytag294)

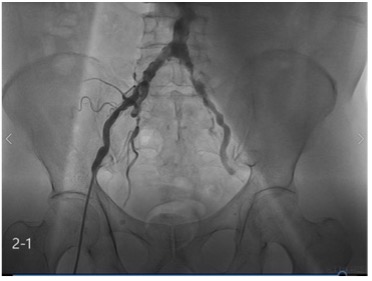

Supplement: ytag294_Supplementary_Data [file ytag294_supplementary_data.zip › Supplementary Figure 1.jpg]

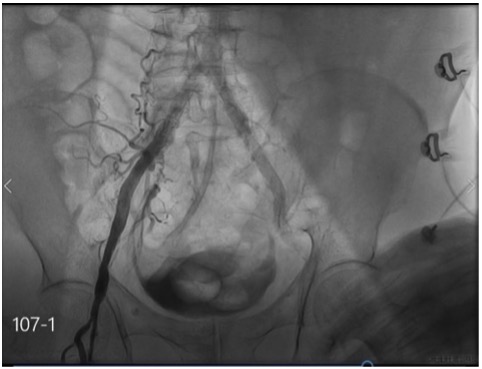

Supplement: ytag294_Supplementary_Data [file ytag294_supplementary_data.zip › Supplementary Figure 2.jpg]

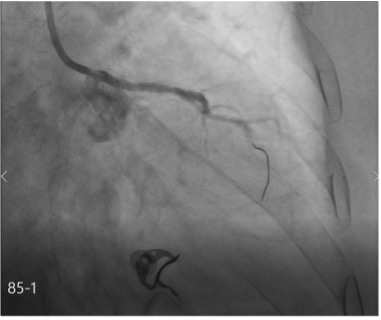

Supplement: ytag294_Supplementary_Data [file ytag294_supplementary_data.zip › Supplementary Figure 3.jpg]

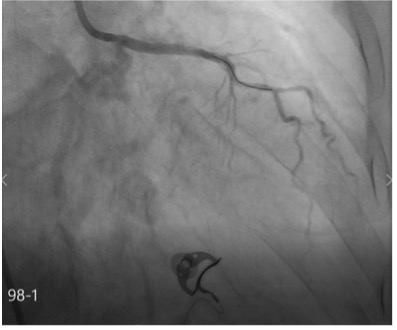

Supplement: ytag294_Supplementary_Data [file ytag294_supplementary_data.zip › Supplementary Figure 4.jpg]
